# Evolution of odorant receptor repertoires across Hymenoptera is not linked to the evolution of eusociality

**DOI:** 10.1098/rspb.2024.1280

**Published:** 2024-09-25

**Authors:** Shubham Gautam, Sean McKenzie, Julian Katzke, Francisco Hita Garcia, Shûhei Yamamoto, Evan P. Economo

**Affiliations:** ^1^ Biodiversity and Biocomplexity Unit, Okinawa Institute of Science and Technology Graduate University, 1919-1 Tancha, Onna-son, Okinawa 904-0495, Japan; ^2^ Oxford Nanopore Technologies, Oxford, UK; ^3^ Center for Integrative Biodiversity Discovery, Museum für Naturkunde Invalidenstraße, Berlin 10115, Germany; ^4^ Hokkaido University Museum, Hokkaido University, Kita 10, Nishi 8, Kita-ku, Sapporo 060-0810, Japan

**Keywords:** odorant receptors, 9-exon, eusociality, Hymenoptera, neuroanatomy, antennal lobes

## Abstract

Communication is essential for social organisms. In eusocial insects, olfaction facilitates communication and recognition between nestmates. The study of certain model organisms has led to the hypothesis that odorant receptors are expanded in eusocial Hymenoptera. This has become a widely mentioned idea in the literature, albeit with conflicting reports, and has not been tested with a broad comparative analysis. Here we combined existing genomic and new neuroanatomical data, including from an approximately 100 Myr old fossil ant, across a phylogenetically broad sample of hymenopteran lineages. We find no evidence that variation in the size and evolutionary tempo of odorant receptor repertoires is related to eusociality. Post hoc exploration of our data hinted at loss of flight as a possible factor shaping some of the variation in OR repertoires in Hymenoptera. Nevertheless, our analyses revealed a complex pattern of evolutionary variation, and raise new questions about the ecological, behavioural and social factors that shape olfactory abilities.

## 1. Introduction

Communication is essential to enhance cooperation and reduce aggression between individuals of a social group. Owing to the additional need of recognizing and communicating with nestmates, sensing and discriminating cues is thought to be of greater importance for eusocial insects. Although a variety of cues (e.g. visual and auditory) can be used for communication, cuticular-hydrocarbons (CHCs) in particular seem to be predominant and ubiquitous among eusocial Hymenoptera [1–4]. As a result, bees, ants and some wasps have been extensively used as model organisms to understand how chemical communication facilitates eusocial life-history strategies [5–9]. Application of recent advances in genomics and imaging in this field has resulted in a large body of knowledge characterizing the molecular and neuroanatomical bases of chemosensation [10,11].

To detect chemical signals and cues, insect antennae and some other body parts are covered with olfactory sensilla containing the dendrites of the olfactory and gustatory receptor neurons. These neurons house chemoreceptors that are largely classified into gustatory receptor (GR), ionotropic receptor (IR) and odorant receptor (OR) families [12]. Particularly, odorant receptors are some of the most rapidly evolving genes and make up one of the largest gene families in insect genomes [12–14]. The receptor proteins in the OR gene family bind different odorants with varying levels of affinity and the signal is then transmitted through olfactory receptor neurons’ (ORN) axons to the primary olfactory centre of the brain, the antennal lobes. Here, olfactory discrimination of chemical cues is achieved through the ‘one neuron–one receptor’ rule. Although some exceptions to this rule are known from *Drosophila*, mosquitoes and orthopterans [15–20], generally each ORN expresses only one odorant receptor and all ORNs that express the same receptor extend their axons to a single structural unit of the antennal lobes, called a glomerulus [21,22]. Moreover, recent work with ants highlighted that even with widespread OR co-expression, each ORN ultimately produces a single or very few functional receptors [5].

Consequently, the antennal lobes in most insect brains consist of a species-specific number of glomeruli that tightly correlates with the number of OR genes [23,24]. Variation in OR gene repertoire and subsequently in glomeruli number is thought to be largely shaped by species chemosensory ecology. Given the importance of olfactory communication in eusocial insects, it was thought that OR gene repertoire variation should reflect adaptations to a social lifestyle [25–28]. Therefore, reliance on olfactory communication in eusocial Hymenoptera for tasks like nestmate recognition, caste and species discrimination, and reproductive signalling is hypothesized to favour increased copy number of OR genes to facilitate social communication. We refer to this as the ‘eusociality hypothesis’. Accordingly, early genomes from ants and bees revealed some of the largest OR repertoires known for any insects [26,27,29], giving rise to the hypothesis that greatly expanded OR repertoires may be associated with eusociality in Hymenoptera [25–30]. Soon after, however, exceptions appeared when the genomes of some solitary wasps like *Nasonia vitripennis* and *Microplitis demolitor* revealed higher number of OR genes in comparison to *Apis mellifera* [31,32].

Later, the 9-exon subfamily of OR genes garnered much attention in the context of evolution of eusociality due to its striking expansion in eusocial Hymenoptera [23,24,33–35]. In ants, 9-exon ORs are exclusively expressed in and near the basiconic sensilla of females and are thought to respond to cuticular hydrocarbons [36,37]. The neurons expressing these receptors innervate a specific cluster of glomeruli, called the T6 cluster [22,24,33]. Similarly, a large cluster of glomeruli called the TB cluster in the antennal lobe of wasps is thought to be homologous to the T6 cluster in ants [38]. The TB/T6 does not share local connections with other glomeruli clusters and its projections to the higher order centres are segregated [39]. Overall, based on these observations, it was proposed that the TB/T6 cluster represented a distinct and specialized olfactory subsystem for processing CHCs thereby facilitating nestmate recognition and cooperation [40,41].

In support of the eusociality hypothesis, recent studies proposed that lineages representing independent evolution of eusociality may have independently evolved expanded 9-exon subfamily of ORs and its corresponding T6 glomeruli [23,24,40]. However, this hypothesis has come into question from at least two indirect lines of evidence. First, the exclusive link between sensing CHCs and T6 glomeruli is questionable. Sharma *et al*. reported that the basiconic sensilla (innervating the T6 cluster) in ants not only respond to CHCs but also respond to general odorants [42]. Furthermore, while 9-exon ORs seem to respond more strongly to CHCs, Slone *et al*. showed that CHCs are not exclusively detected by the 9-exon ORs. Several other receptors outside the 9-exon subfamily also respond to CHCs [34], which undermines the hypothesis of a specialized subsystem for social recognition. Second, Karpe *et al*. reported at least one solitary bee with (*Habropoda laboriosa*) equally large 9-exon OR repertoire as compared with eusocial honeybees [43]. Brand & Ramírez showed that eusocial stingless bees have rather reduced 9-exon repertoires [44], raising questions about the validity of the association between expanded 9-exon ORs and the evolution of eusociality.

Earlier studies had reported expansions in several other subfamilies of the OR sub-genome in Hymenoptera. For example, ants, bees and wasps were reported to have lineage-specific expansions in the U, J and F subfamilies, respectively [35]. Similarly, in addition to the 9-exon subfamily, L, T, H, E and V subfamilies were also reported to be expanded in *Polistes* wasps [23], and the L subfamily in apoid wasps [45]. Unlike the 9-exon subfamily and its corresponding TB/T6 cluster, the rest of the OR sub-genome and the other half of the antennal lobe have received little attention in comparative analyses. Currently, there is no information on whether different OR subfamilies are functionally specialized, other than the 9-exon ORs that are thought to be a specialized sub-system for processing cuticular hydrocarbons. Furthermore, whether receptors from these subfamilies feed to a specific cluster of glomeruli in the antennal lobe has not been established. In the absence of such information, analysing specific clusters other than the TB/T6 cluster in this framework is challenging. Nevertheless, comparisons between the glomeruli number in the TB/T6 cluster and the rest of the antennal lobe have recently been studied in some vespid wasps [46].

**Figure 1.**
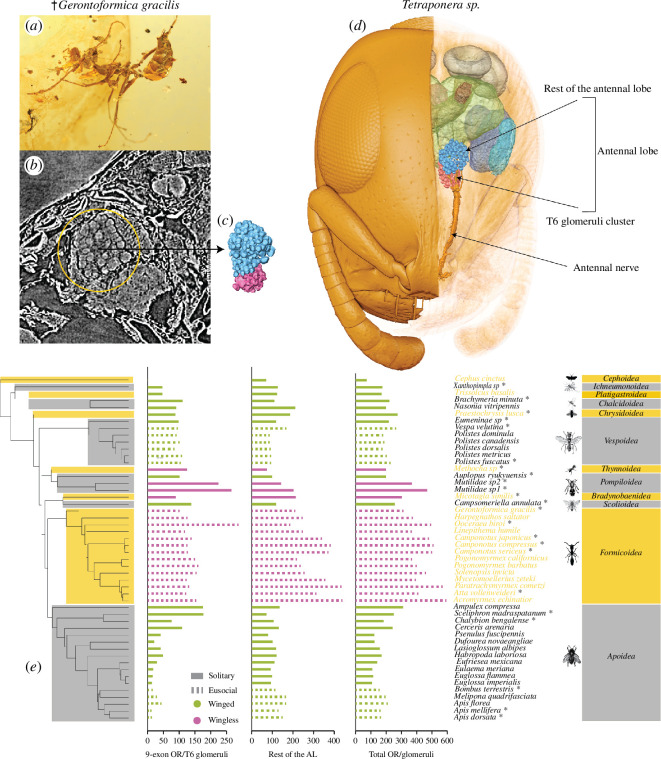
Evolution of odorant receptor number in Hymenoptera. (*a*) Approximately 100 Myr old fossil specimen of *Gerontoformica gracilis*. (*b*) Two-dimensional slice from micro-CT imaging showing the exceptionally preserved glomeruli in the antennal lobes of the brain of the fossil. (*c*) 3D reconstructed glomeruli from the left antennal lobe of the fossil ant. The T6 cluster of the glomeruli is coloured pink in the reconstruction. (*d*) 3D reconstructed left hemisphere of the brain of *Tetraponera* sp. highlighting different neuropils and differentiation of individual glomeruli. The T6 cluster (coloured in pink) forms a hemi-lobe with the partially reconstructed sensory tract approaching the cluster from the periphery of the antennal lobe. (*e*) Distribution of 9-exon (left), rest of the antennal lobe (centre) and total OR (right) repertoires plotted on the phylogeny. Dotted bars represent eusocial taxa and solid bars are for solitary taxa. Bars coloured in green are for taxa with winged females and in purple are for taxa with wingless females. Taxon names marked with asterisks represent the set of taxa that qualified for glomeruli-only analyses.

Despite extensive research on ORs and sociality, independent tests of the association between eusociality and expanded OR numbers have been limited to smaller phylogenetic scales and many lacked key solitary outgroups for appropriate comparisons. A broader comparative analysis filling these crucial phylogenetic holes has been missing, as stressed by a recent review on this topic [6]. Here, we reconstruct the evolutionary dynamics of odorant receptor repertoire in Hymenoptera to answer (i) whether ants and *Polistes* wasps stand out in having expanded 9-exon, non-9-exon and total OR repertoires, and (ii) whether evolution of eusociality is associated with variation in the size and the rate of evolution of these repertoires. Taking advantage of the strong correlation between OR gene number and the number of glomeruli in the antennal lobes, we combined existing genomic and new neuroanatomical data to achieve our desired sample. We also added neuroanatomical data from an approximately 100-million-year-old stem group ant fossil with remarkable soft tissue preservation of the brain (figure 1*a,b*) [47], to test whether the purported expansion of OR repertoires mapped exclusively to the crown group ants or the most recent common ancestor of all ants. Finally, we conduct a post hoc test of an alternative ecological hypothesis for guiding future research in this field.

## 2. Results

### (a) Effect of sequencing technology: short-read versus long-read-based genomes

One of the potential sources of uncertainty in our study stems from combining three different sources of data, i.e. gene counts from (i) short read genomes, (ii) long read genomes, and (iii) glomeruli counts from the antennal lobes in the brain. It is possible that the sequencing technology used to sequence the genomes have systematic effects on detecting ORs in the genome [48]. To test for such an effect, where we had data for all three sources, we assessed the scatter of data points from the expected 1 : 1 gene to glomeruli relationship and estimated correlation coefficients for all different sources of data (electronic supplementary material, figure S1). These data showed strong correlations between all methods, allowing us to combine different data sources to build our response variable. Nevertheless, for robustness checks, analyses performed on alternate response variables where gene numbers were prioritized instead of glomeruli counts and the analysis of glomeruli-only subset of our dataset revealed consistent patterns of no effect of eusociality on OR repertoires (electronic supplementary material, table S1).

### (b) Estimation of evolutionary shifts in OR repertoires

**Figure 2.**
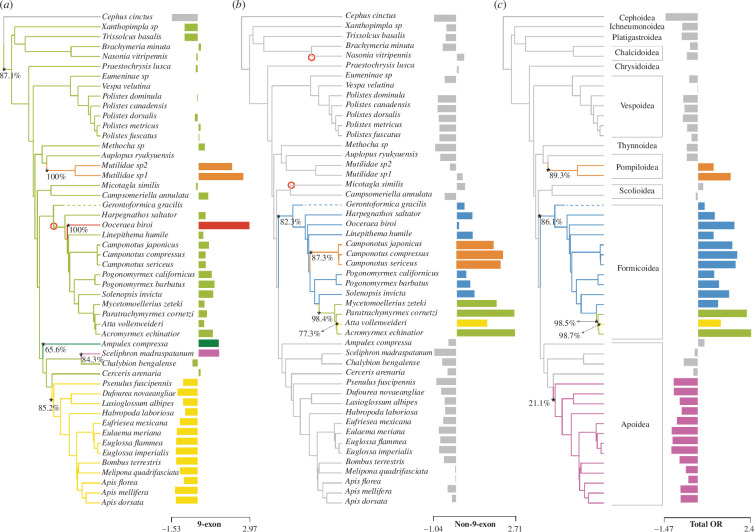
Model-based inference of macroevolution of odorant receptor repertoires. Estimated evolutionary shifts in the size of (*a*) 9-exon, (*b*) non-9-exon and (*c*) total odorant receptor repertoires under multi-regime Ohrenstein–Uhenbeck models inferred with L1OU package in R. Colours reflect different evolutionary optima associated with evolution on different regions of the phylogeny. Nodes with significant shifts are marked with an asterisk with annotations of estimated bootstrap supports. Open red circles represent significant positive shifts that were estimated in at least one of the evaluated alternative models.

We detected 6, 4 and 5 shifts in the repertoire size of 9-exon, rest of the AL and total OR, respectively. In most of the evaluated models, ants and Polistes wasps did not have any significant shifts in the number of 9-exon receptors (figure 2*a*). Although one of the evaluated models did estimate a significant positive shift on the branch leading to the most recent common ancestor (MRCA) of the crown group ants (indicated by the red open circle in figure 2*a*). One of the consistent shifts across all models for 9-exon ORs was estimated for *Ooceraea biroi*, highlighting this clade as an outlier in comparison to other ants. Mutillid wasps stood out for having significant expansions in the number of 9-exon ORs. Another significant but negative shift was detected on the branch leading to the MRCA of Anthophilla (bees) + *Psenulus*, indicating a secondary reduction in the repertoire size of 9-exon ORs in bees.

The shifts in the number of non-9-exon ORs estimated at the branch leading to the MRCA of all ants (including the fossil *Gerontoformica*) and other clades within Formicoidea were consistent across all evaluated models (figure 2*b*). Two additional shifts in the evaluated set of alternative models were estimated for *N. vitripennis* and *Micotagla similis* as indicated by the open red circles in figure 2*b*.

For total OR repertoires, significant positive shifts were estimated for the branches leading to mutillid wasps and ants (figure 2*c*). Another positive and one negative shift was estimated within the ants among the Attines. The MRCA of Anthophilla (bees) + *Psenulus* was estimated to have a negative shift in the number of total ORs, however, with much lower (approx. 20%) bootstrap support. Results from fitting a birth–death model of evolution using CAFE software [49] recovered similar shifts along the exact same branches, along with more detailed changes recovered along the internal nodes of the tree (electronic supplementary material, figures S4–S7).

### (c) Size and evolutionary tempo of OR repertoires are seemingly independent of the evolution of eusociality in Hymenoptera

**Figure 3.**
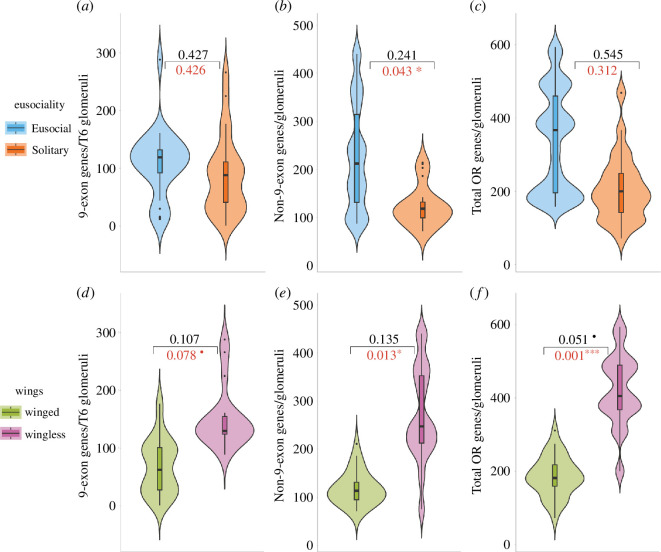
Evolutionary correlation between odorant receptor number and eusociality and winglessness. Box and violin plots for the number of 9-exon (a and d), non-9-exon (b and e) and total ORs (c and f) with estimated *p* values from the RRPP in black and a regular phylogenetic ANOVA in red. Top row shows a lack of any clear effect of eusociality on OR repertoire sizes; however, the bottom row highlights a potential trend of expanded OR repertoires for wingless females although not statistically significant with this dataset. **p < 0.001; *p < 0.05; • *p* < 0.1; n.s. *p* > 0.1.

The distribution of the number of odorant receptors against the phylogenetic tree hinted at no clear effect of eusociality on OR repertoires as closely related eusocial taxa and their solitary outgroups showed little variation in OR number (figure 1*e*). Statistical tests of this relationship using residual randomizations using permutations (RRPP) ANOVA failed to detect any association between eusociality and the size of 9-exon, non-9-exon and total OR repertoires (figure 3*a–c*, respectively).

**Figure 4.**
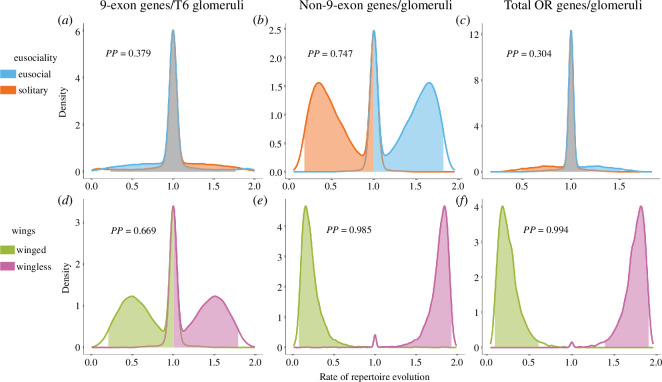
Rates of evolution of odorant receptor number and evolutionary correlates. Estimated posterior distributions of the rate parameter using multiple state-specific rates of continuous character evolution (MuSSCRat) for (*a,d*) 9-exon, (*b,e*) non-9-exon and (*c,f*) total OR repertoires of social (blue) and solitary (orange) in the top row and winged (green) and wingless (purple) states at the bottom. These results show no effect of eusociality on the rates of 9-exon and total OR repertoire evolution as the distributions widely overlap. Plots in the bottom row show mixed support for the effect of loss of wings on the rates of 9-exon repertoires, but clear differences for the rates of non-9-exon and total OR repertoires between winged and wingless lineages. Posterior probabilities (PP) of the state-dependent models are reported within each plot.

After accounting for background rate variation with multiple state-specific rates of continuous character evolution (MuSSCRat), we found that the posterior distributions of rates of 9-exon and total OR repertoire evolution did not differ between eusocial and solitary states (figure 4*a–c*). However, there was mixed support for the effect of eusociality on the rates of non-9-exon repertoires, with social taxa tending to have higher rates in comparison to solitary Hymenoptera. Posterior estimates of the rates were not strongly affected by different priors on the number of rate shifts. For eusociality character, posterior probabilities of state-dependent rates for total OR repertoires ranged from 0.39 to 0.44.

### (d) Size and evolutionary tempo of OR repertoires might be associated with the evolution of flightless females in Hymenoptera

Testing the effect of flightlessness remains a post hoc analysis with our dataset and it is presented here as an exploratory step to provide a new direction for future research in the field. Bar plot for non-9-exon and total OR repertoires coloured according to the presence or absence of flightless females hinted at a pattern of elevated total OR repertoires in flightless taxa but no effect on 9-exon repertoires (figure 1*b*). This pattern found some support with a regular phylogenetic ANOVA as well as RRPP ANOVA, which showed wingless taxa tended to have elevated OR repertoires, although statistically non-significant (electronic supplementary material, table S1, figure 3*d–f*).

After accounting for background rate variation using MuSSCRat model, posterior distributions of state-dependent rates for 9-exon ORs showed mixed results for the difference between winged and wingless females (figure 4*d*). However, for non-9-exon and total OR repertoires, wingless females showed clearly elevated evolutionary rates in comparison to winged females (figure 4*e, f*), suggesting strong influence of loss of wings on rates of evolution of total and non-9-exon OR repertoires. For loss of wings, posterior probabilities of state-dependent rates were not strongly affected by different priors on the number of rate shifts. Posterior probabilities for state-dependent rates of total OR repertoires ranged from 0.93 to 0.97.

## 3. Discussion

By combining genomic and neuroanatomical data and building on previous datasets, our study sheds light on the evolution of odorant receptor repertoires in Hymenoptera over the past approximately 250 million years. Our findings contrast with established results in the field and cast doubt on the eusociality hypothesis while revealing new complexity in the macroevolution of OR repertoires. Based on the patterns observed in our dataset, we provide an alternative post hoc hypothesis that should be tested more rigorously in future studies. Overall, our results suggested that evolution of flightless females, not eusociality, may be associated with elevated tempo and size of OR repertoires.

### (a) Evolutionary shifts in OR repertoire size

We aimed to understand when and where the evolutionary shifts in the repertoire size of 9-exon, non-9-exon and total OR repertoires occurred on the Hymenoptera tree. Our results stand in contrast to the patterns reported in recent studies. In two separate studies, ants and *Polistes* wasps were reported to have massively expanded 9-exon OR number [23,24]. Our analysis failed to detect any significant positive shifts associated with the *Polistes* clade and only one of the eight top alternative models estimated a significant shift specific to the MRCA of crown group ants. Potential reasons behind the discordance of previous studies from our results may be due to those studies including a combination of a small number of solitary outgroups (*Microplitis* and *Nasonia*) that turned out to have unusually small repertoires for solitary wasps, as well as a focus on an outlier in the number of 9-exon ORs, namely, the clonal raider ant (*Ooceraea biroi*). Although it is possible that expanded 9-exon ORs are common to Dorylinae, as *Eciton burchellii* was also shown to have similarly expanded 9-exon repertoires [50], which may be associated with dietary specialization in this clade.

Another significant but negative shift was estimated for the branch leading to the MRCA of Anthophila+Psenidae clade. This shift suggested a secondary reduction in the 9-exon OR repertoires in this clade. Such secondarily reduced 9-exon repertoires in bees and the lack of solitary apoid and aculeate wasps in comparative studies might have caused the inference of apparent expansions of 9-exon repertoires in ants and *Polistes* wasps. Observed reduction in the 9-exon OR repertoires is in concurrence with a recent analysis, which included appropriate apoid wasps as the solitary outgroups of social bees [45]. This reduction is hypothesized to be linked to the shift from a predatory lifestyle to obligate herbivory in this clade. Similar loss of odorant receptors has been linked to the evolution of herbivory in drosophilids [51].

In contrast to previous reports, our results suggest a surprising pattern where the MRCA of all ants may have undergone expansions in the non-9-exon ORs, followed by several clade-specific expansions and reductions (figure 1*b*). Unfortunately, without new genomes it is not possible to determine the specific OR subfamily corresponding to these expansions; however, it is likely that previously reported expansions in the U subfamily of ORs may be responsible for this pattern [35]. Future genomes from mutillids and other solitary wasps may be useful in pinpointing the specific subfamilies underlying these expansions in ants.

Overall, ants and *Polistes* wasps do not seem to have particularly expanded 9-exon OR repertoires. Non-9-exon repertoires, however, seem to have undergone several clade-specific shifts in ants. Unfortunately, choice of an outlier ant, secondarily reduced 9-exon repertoires in bees and unavailability of apoid and aculeate wasp genomes may have led previous studies to the conclusion that ants and Polistes wasps seem to have especially expanded 9-exon repertoires [23,24].

### (b) OR expansion and eusociality

Ever since the first genomes of social insects became available, a link between expansions of odorant receptor repertoires and eusociality has been hypothesized [26,31,35] and subsequently contested [43,52]. Later, specifically, the 9-exon clade of OR genes and its corresponding glomeruli in the antennal lobes of the brain were invoked as a specialized subsystem for kin recognition and are therefore thought to be associated with the evolution of eusociality in Hymenoptera [23,24,40,41]. However, independent tests of this hypothesis have reported mixed results [43,44]. Our analyses with an updated and phylogenetically informed dataset revisited the eusociality hypothesis and failed to find any support for an association between the size and rates of OR repertoire evolution and eusociality. Moreover, if 9-exon ORs indeed specialized in responding to CHCs, it would be expected that taxa with expanded 9-exon repertoires would also harbour more diverse and/or complex CHC repertoires. However, a recent meta-analysis reported that the phenotypic complexity of cuticular hydrocarbons does not differ between eusocial and solitary taxa [53]. This trend is in accordance with our results showing no effect of eusociality on 9-exon as well as other odorant receptor repertoires. Our study by no means excludes a role of OR genes in social communication, but it does suggest that this is not reflected in gene or glomeruli numbers alone.

It is possible that eusociality does not lead to systematically larger OR repertoires, but it might still affect the rate of evolution of these repertoires. Therefore, we tested the effect of eusociality on the rate of evolution of OR repertoires and failed to detect any differences between the rates of 9-exon as well as total OR repertoires of social and solitary lineages. However, for non-9-exon ORs a trend for higher repertoire size and rates was observed albeit statistically non-significant with this dataset. Taken together, these patterns suggest that eusociality does not result in the evolution of increased size or evolutionary rates of odorant receptor repertoires.

### (c) Flightless females and potentially elevated size and tempo of total OR repertoires

After discovering a lack of an effect of eusociality, we carefully assessed the patterns in our data and performed a post hoc test of the effect of loss of flight in females on the size and evolutionary rates of OR repertoires. Our taxon sampling covered four independent losses of winged flight in hymenopteran females, out of which at least three (i.e. Formicoidea, Bradynobaenidea and Mutilidae) showed expansions in total OR repertoire. We were unable to sample a winged Thynnoidea female to confidently assess OR expansions in species that secondarily lost winged flight in this clade. Overall, our post hoc analysis suggested that secondary loss of wings may be associated with elevated rate of evolution and size of non-9-exon and total OR repertoires; however, the tempo and size of 9-exon OR repertoires seem unaffected.

Odorant receptors evolved from the gustatory receptor gene family in insects [54,55] and are likely to be absent in non-insect arthropods [56,57]. Evolution of ORs in insects was hypothesized to coincide with terrestriality in insects, but recent analysis suggested an alternative hypothesis positing that insect ORs evolved in response to winged flight [58]. This hypothesis was later not supported with an updated analysis which showed that ORs evolved before the evolution of winged flight in insects, possibly as an adaptation to terrestriality [59,60]. Our results reveal a complex pattern that is currently poorly understood and provide preliminary indications that expansions in OR repertoires may be linked to terrestriality. These results suggest that secondary loss of wings results is not only directional evolution of OR repertoires towards larger sizes but also a faster rate of OR repertoire evolution for wingless lineages in comparison to lineages with winged females. Such a pattern of OR repertoire evolution might be in response to a more diverse and stable non-volatile chemical environment on ground as opposed to in air, where volatile compounds are likely to be extremely diluted and disperse sooner and more often. As a result, winged insects may require fewer but more sensitive ORs to optimally navigate their olfactory environment. However, we stress that this analysis is merely an exploratory step to generate a new hypothesis which along with other potential drivers such as dietary ecology, trade-offs between vision and olfaction, etc., should be tested rigorously in future studies.

## 4. Conclusions

There is a great deal left to be learned about the evolution of OR repertoires in insects, but the results reported here are suggestive of broader patterns driving the evolution of this crucial trait. Ants and *Polistes* wasps are not special in having expanded repertoires of 9-exon odorant receptors. Choice of an outlier lineage of ants and secondary reductions of 9-exon ORs in bees may have led previous studies to infer that ants and *Polistes* wasps seem to have extraordinary expansions in these ORs. In contrast to a widely known hypothesis, the evolutionary dynamics of odorant receptor genes seem to be largely independent of the evolution of eusociality. Secondary loss of flight in hymenopteran females could be associated with elevated rates of evolution and expansions in non-9-exon and total OR repertoires. The forces shaping the evolution of the 9-exon OR subfamily, and odorant receptors in general appear more complex than previously reported, and will require more work to elucidate.

## 5. Material and methods

### (a) Taxon sample

Hymenoptera is a highly diverse insect order and possesses several remarkable characteristics, notably the striking repeated evolution of eusociality. We sampled a set of taxa covering three independent evolutionary transitions to eusociality across the hymenopteran tree. First, we collated published genomic and transcriptomic data on total and 9-exon OR gene repertoires as well as the number of glomeruli in the antennal lobe (electronic supplementary material, table S2) [7–9,22–24,26–29,31,39,40,43–45,61–69]. However, the methods underlying these data differ and following that also the reliability of the counts. Counts originated from less reliable short read as well as relatively more reliable long read genomes. Additionally, studies with short read genomes report counts with and without pseudogenes. It is possible that some functional genes may have been mischaracterized as pseudogenes due to sequencing and other downstream errors. Based on the patterns we observed in the few taxa for we had data from all three sources (i.e. glomeruli count, gene counts from short read genomes and counts from long read genomes), we applied the following general rule to create a response variable for our analyses. Wherever available, we used glomeruli counts as the priority followed by gene counts from long read genomes, and then counts including pseudogenes from short read genomes. We did this as the count including pseudogenes tracked glomeruli count more closely as opposed to the number of putatively functional genes only (electronic supplementary material, figure S1). Additionally, to rule out any unknown effect of mixing data from different sources, we created and analysed two alternate response variables. First, where we prioritized gene counts over glomeruli counts (*n* = 50), and second, a dataset limited to glomeruli counts (*n* = 23) which we call the ‘glomeruli-only’ dataset. The glomeruli-only dataset contained all species for which glomeruli counts were available regardless of whether the gene counts were available. We did not separately analyse the ‘genes-only’ dataset as that would exclude all the key groups that we added with our neuroanatomical dataset and would be equivalent to analysing the same datasets as previous studies.

To the collated gene counts, we added new neuroanatomical data on glomeruli counts from vouchered museum specimens representing several social and solitary hymenopteran lineages. These taxa were specifically sampled for filling key phylogenetic holes such as Scolioidea, Bradynobaenidae and Mutillidea representing the solitary outgroups of ants and bees. We also sampled a solitary vespoid and two apoid wasps to recreate a more accurate history of the evolution of this key trait. Additionally, several Euglossini species are known to be facultatively eusocial. In our dataset, as we only had four species in the ‘facultative eusociality’ state, we decided to assign a solitary state to these taxa instead. We did this to avoid making inferences about the effect of facultative eusociality on OR repertoires based on a small sample size. We also tested the effect of treating these species as eusocial and found similar trends in our results. Overall, we gathered relevant data for 62 taxa with an overrepresentation of ants in this dataset. Especially, attine ants were over-represented in our dataset. For further analyses, we dropped eight attines and *Camponotus floridanus* to achieve a more balanced phylogenetic sample. Species for which both glomeruli and gene counts were available were prioritized to be included in the response variable.

### (b) Neuroanatomy

Field-collected female wasps were fixed and stored in 99% ethanol. We decapitated the specimens and stained the entire head with 0.5% phosphotungstic acid (PTA) solution for at least 14 days to achieve high contrast within the antennal lobes. Each head was then transferred into a pipette tip filled with 99% ethanol, to be placed as a specimen holder inside the scanner. All micro-CT scans were performed using a Zeiss Xradia 510 Versa three-dimensional (3D) X-ray microscope operated by the Zeiss Scout-and-Scan Control System software (v. 11.1.6411.17883). 3D reconstructions of the resulting scan projection data were done with the Zeiss Scout-and-Scan Control System Reconstructor (v. 11.1.6411.17883) and saved in a TXM file format. Image acquisition settings such as voltage, power and exposure time were calibrated for each specimen to achieve maximum resolution and enhanced image contrast for further analyses. Details of methods relating to the imaging of the fossil specimen are listed in a recent paper describing the fossil [47].

We used the resulting stacks of two-dimensional images to reconstruct and manually segment each glomerulus along one of the three focal planes in Amira. The T6 or the TB cluster as it is known for wasps, forms a hemi-lobe medially and is located ventrally in wasps but anteriorly in ants (figure 1). Each glomerulus was assigned to fall within or outside the T6/TB cluster following the innervating input tracts through image stacks. When innervating input tracts could not be confidently resolved, we used visible clustering and separation of glomeruli to group them into one of the clusters. We counted T6/TB and total number of glomeruli in the entire antennal lobe once during the segmentation process and once with the 3D reconstructed glomeruli. There were no differences between the two counts.

### (c) Phylogenetic tree

To construct a phylogenetic tree for our taxon sample, we used the dated tree from Peters *et al*. [70] as the main backbone tree [70] onto which several other clades were grafted using the phytools package in R [71]. The ant clade was extracted from Economo *et al*. [72], Anthophila clade from Cardinal *et al*. [73] and *Polistes* clade was traced from Sheehan *et al*. [74] using the Phylosketch library in R. When an exact species match between our sample and species on the trees used here was not found, we replaced our taxon with the closest possible taxon available in the trees when possible to do so without altering the topology (for example, replacing one member of a monophyletic group with another one). Additionally, we manually added the fossil *Gerontoformica gracilis* such that it branched at 125 Myr and went extinct at 100 Myr. Divergence time estimates for stem-group ants are uncertain, but we used the dates based on the conservative estimates of [75]. We believe that any small differences in the placement of the fossil branch will not significantly affect our results. Overall, we constructed a dated tree with 50 tips to run our final statistical analyses.

### (d) Statistical analyses

To estimate evolutionary shifts in the size of OR repertoires on individual branches along the Hymenoptera phylogeny, we used least-squares multi-regime Ornstein–Uhlenbeck (OU) modelling using the L1OU package in R [76]. This method allows estimation of evolutionary shifts between selective regimes without an *a priori* hypotheses regarding the location of shifts on the tree. For L1OU analysis, which is limited to ultrametric trees, we manually extended the branch containing the fossil to *t* = 0. To ascertain the effect of treating *Gerontoformica* as an extant taxon on our tree, we ran a similar analysis with the original branch length for the fossil using Surface package [77], which allows inputting non-ultrametric trees. OR gene annotations based on transcriptomes may be considered incomplete, to ascertain that gene counts based on transcriptomes did not affect these results, we estimated evolutionary shifts on the tree excluding these cases (electronic supplementary material, figures S2 and S3). Model selection was performed using AIC to be able to compare the two methods. We evaluated the best fitting model as well as alternative plausible models with comparable fit (models with AICc scores within two of the best-fit model) to identify significant shifts in repertoire size of 9-exon, rest of the AL and total OR. We rejected models with unrealistic parameter estimates as model selection with AICc can favour overfitting. While the OU model is based on continuous valued trait, gene numbers are discrete valued evolving through birth–death processes. Although the evolution of an integer-valued trait should be similar to a continuous trait when numbers are large (as is the case here), we fitted a birth–death model of evolution using a method specifically designed for gene family evolution (CAFE) to reconstruct gene family size at the internal nodes and identify branches with expansions and/or contractions that are significantly different from expectations under random evolution [49,78].

To evaluate statistical differences in 9-exon and total OR repertoire size among social and solitary Hymenoptera, we aimed to use a simple phylogenetic ANOVA. However, when the grouping variable or the discrete character of interest shows aggregation along the phylogeny, the standard phylogenetic ANOVA suffers from reduced statistical power and incorrect parameter estimates [79]. To overcome this, we used the modified method of RRPP which display higher power than standard phylogenetic ANOVA [80]. RRPP produces sampling distributions for ANOVA statistics by repeatedly randomizing residuals from a null model and uses them to estimate coefficients for the alternative model. We performed independent RRPP ANOVAs with 10 000 iterations each on 9-exon, non-9-exon and total OR numbers with sociality state as the explanatory variable.

We tested the effect of eusociality on the tempo of evolution of 9-exon and OR repertoires by employing a recently proposed method called the MuSSCRat. This method controls for erroneous attribution of the presence of any rate difference to the effect of the studied variable by introducing background rate variation in the model [81]. MuSSCRat allows inferring the impact of discrete traits on evolutionary rates in the presence of background rate variation. The MCMC models were run for 100k generations with 10% burnin rate in Revbayes [82]. We repeated this analysis with different priors on the expected number of shifts (10 and 20) to evaluate its effect on posterior estimates of the state-dependent rate parameters.

## Data Availability

All R and RevBayes programming code, raw data on gene and glomeruli counts necessary to evaluate the conclusions of this study, and all CT-scans used for glomeruli counts are available as supplemental information at Dryad repository [83]. Supplementary table and figures are available as electronic supplementary material online [84].
